# Inequity in insurance coverage for prescription drugs in New Brunswick, Canada

**DOI:** 10.17269/s41997-022-00639-3

**Published:** 2022-04-29

**Authors:** Busola Ayodele, Elaine Xiaoyu Guo, Arthur Sweetman, G. Emmanuel Guindon

**Affiliations:** 1 Centre for Health Economics and Policy Analysis, McMaster University, CRL Building, Room 229, 1280 Main Street West, Hamilton, ON L8S 4K1 Canada; 2 Department of Economics, University of Toronto, Toronto, ON Canada; 3 Department of Economics, McMaster University, Hamilton, ON Canada; 4 Department of Health Research Methods, Evidence, and Impact, McMaster University, Hamilton, ON Canada

**Keywords:** Drug insurance, Socioeconomic factors, Health equity, Canada, New Brunswick, Assurance médicaments, facteurs socioéconomiques, équité en santé, Canada, New Brunswick

## Abstract

**Objectives:**

To describe the extent to which New Brunswick residents reported having drug insurance coverage supplementary to Canadian Medicare; to examine associations between socioeconomic and demographic characteristics, health status, language identity, and having reported such coverage; and to document any changes in coverage associated with the introduction of the New Brunswick Drug Plan in 2014.

**Methods:**

We used repeated cross-sectional data for New Brunswick from eight cycles of the Canadian Community Health Survey from 2007 to 2017 and undertook logistic regression analysis.

**Results:**

We found statistically significant, substantial and policy-relevant socioeconomic differences in the reporting of prescription drug insurance coverage among those 25–64 years and those ≥ 65 years of age, and an increasing reliance on private drug insurance over time. We found that individuals in the second decile of household income were particularly vulnerable to reporting neither public nor private drug coverage. The introduction of the New Brunswick Drug Plan in 2014 does not appear to have led to increased public drug coverage; however, from 2014, the decreasing trend in public drug coverage appears to have ceased. Those who reported lower health status usually had lower odds of reporting private drug coverage but higher odds of reporting public drug coverage. Driven by differences in private coverage, we found that relative to anglophones, francophones were less likely to report any drug coverage.

**Conclusion:**

Our findings emphasize the shortcomings of drug insurance systems such as that introduced in New Brunswick and substantiate calls for a universal drug program. New Brunswick’s increasing reliance on private drug insurance is of concern and warrants additional research.

## Résumé

**Objectifs:**

Décrire la mesure dans laquelle les résidents du Nouveau-Brunswick ont déclaré avoir une couverture d’assurance médicaments supplémentaire au régime public d’assurance maladie canadien; examiner les associations entre les caractéristiques socioéconomiques et démographiques, l’état de santé, l’identité linguistique et avoir déclaré une telle couverture; et documenter tout changement de couverture associé à l’introduction du Régime médicaments du Nouveau-Brunswick en 2014.

**Méthodes:**

Nous avons utilisé des données transversales répétées du Nouveau-Brunswick de huit cycles de l’Enquête sur la santé dans les collectivités canadiennes de 2007 à 2017 et avons entrepris une analyse de régression logistique.

**Résultats:**

Nous avons constaté des différences socioéconomiques statistiquement significatives, substantielles et pertinentes en matière de politiques dans la déclaration de la couverture d’assurance médicaments chez les 25 à 64 ans et les 65 ans et plus, et une dépendance croissante à l’égard de l’assurance médicaments privée au fil du temps. Nous avons constaté que les personnes appartenant au deuxième décile du revenu du ménage étaient particulièrement vulnérables au fait de ne pas avoir déclaré d’assurance médicaments publique ou privée. La mise en place du Régime médicaments du Nouveau-Brunswick en 2014 ne semble pas avoir entraîné une augmentation de la couverture publique des médicaments; cependant, à partir de 2014, la tendance à la baisse de la couverture publique des médicaments semble avoir cessé. Ceux qui ont déclaré un état de santé inférieur avaient généralement une cote exprimant la probabilité plus faible de déclarer une assurance médicaments privée, mais plus élevée de déclarer une couverture publique des médicaments. En raison des différences de couverture privée, nous avons trouvé que, par rapport aux anglophones, les francophones étaient moins susceptibles de déclarer une couverture pour les médicaments.

**Conclusion:**

Nos résultats mettent en évidence les lacunes des systèmes d’assurance médicaments comme celui mis en place au Nouveau-Brunswick et justifient les appels en faveur d’un programme universel de médicaments. La dépendance croissante du Nouveau-Brunswick à l’égard de l’assurance médicaments privée est préoccupante et justifie des recherches supplémentaires.

## Introduction

Canada has a universal healthcare system primarily paid for through taxes and public debt that provides insurance coverage, without any cost-sharing, for medically necessary physician and hospital services (including prescription drugs administered in hospitals) to all Canadian citizens and permanent residents (Marchildon et al., 2020). No federal guidelines govern the financing of outpatient prescription drug services. As a result, provincial and territorial governments have independently developed drug insurance programs with varying eligibility requirements and payment arrangements (Canadian Institute for Health Information, 2019). With the exception of Québec, which mandates drug insurance for all its residents, provincial drug insurance plans typically provide some form of catastrophic coverage, most often when costs exceed deductibles set as a proportion of household income. This catastrophic coverage is usually provided in combination with age-based plans, such as the Ontario Drug Benefit (ODB) Program that provides drug coverage to residents under age 25 who do not have private coverage and to all seniors aged 65 and above, or income-based plans such as British Columbia’s Fair PharmaCare (Brandt et al., 2018). Residents who are ineligible for drug coverage under public programs or who are insured under public programs but desire additional coverage can purchase tax-subsidized private drug coverage. Most private drug insurance plans are purchased by employers, unions or professional associations, and provided to eligible employees and members (Brandt et al., 2018; Canadian Life and Health Insurance Association, 2018).

The New Brunswick Prescription Drug Program (NBPDP), introduced in 1975, provides drug coverage to specific beneficiary groups, including low-income seniors, nursing home residents, adults living in licensed residential facilities, social development clients, children with special needs, and children in the care of the Minister of Social Development. The NBPDP is financed, in part, through premiums and cost-sharing which vary depending on eligibility. The NBPDP also provides disease-specific drug coverage (e.g., cystic fibrosis, multiple sclerosis, and HIV/AIDS) (Canadian Institute for Health Information, 2019). The Medavie Blue Cross Seniors Prescription Drug Program, introduced in 1992, is available to New Brunswick seniors who do not qualify for NBPDP coverage (i.e., they do not receive the federal Guaranteed Income Supplement [GIS] or their annual income exceeds the qualifying threshold). This program, which provides the same benefits as those provided by the NBPDP, is premium-based ($125 a month in 2020) with cost-sharing ($15 copay per prescription) with no monthly or annual limit in 2020.

In 2014, New Brunswick launched the “New Brunswick Drug Plan” (NBDP) to provide public insurance to cover the costs of all drugs on the province’s prescription drug formulary. The NBDP’s overarching goal was to ensure that all residents had drug coverage that matched at least the coverage provided by the NBPDP. The initial voluntary phase began in May 2014. Residents, including seniors, who did not have existing drug coverage (private or public) or who had existing drug coverage that did not cover a specific drug that was included in the drug plan formulary or who had reached their early or lifetime maximum for drug coverage could enrol in the NBDP. The province planned to make the plan mandatory to those without drug coverage in April 2015. It was envisaged that the plan costs would be covered by plan members, government, and employers who did not provide a workplace drug plan. Just before launching, the NBDP was lauded as perhaps “the best one in Canada” by Steve Morgan, a prolific researcher of pharmaceutical policy at the University of British Columbia and ardent advocate for national universal pharmacare (Owens, 2014).

**Fig. 1:**
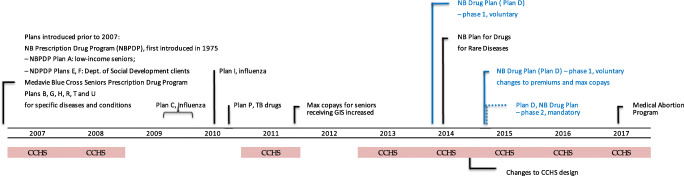
Timeline of key policy changes and Canadian Community Health Survey (CCHS) cycles with drug insurance component. Note. *NB*, New Brunswick; *GIS*, Guaranteed Income Supplement; *Plan B*, cystic fibrosis; *Plan G*, children in the care of the Minister of Social Development; *Plan H*, multiple sclerosis; *Plan R*, organ transplant; *Plan T*, growth hormone deficiency; *Plan U*, HIV/AIDS

In December 2014, the newly elected Liberal government, under pressure from employer organizations such as the Canadian Federation of Independent Business, abandoned making the plan mandatory and requiring employers to contribute to its costs (Glynn, 2017). Premiums for the poorest residents were also reduced from $800 annually to $440 or $200, depending on gross income. Copayments remained unchanged at 30% per prescription with maximum copays set between $5 and $30, depending on gross income, with no maximum annual copayment (Canadian Institute for Health Information, 2017; Government of New Brunswick, 2014). As the mandatory requirement to have prescription drug insurance was eliminated, the New Brunswick government launched a comprehensive review of the NBDP. Although a discussion paper was prepared and a consultation seeking feedback from stakeholders and the public took place, the review was never completed (Department of Health, 2015). Figure 1 presents a timeline of key policy changes in New Brunswick.

Our objectives are to (1) describe the extent to which New Brunswick residents reported having supplementary drug insurance coverage; (2) examine the associations between having reported supplementary drug insurance coverage and socioeconomic and demographic characteristics, health status, and language identity; and (3) learn from New Brunswick’s reform about whether gaps in coverage improved with the introduction of this voluntary pharmaceutical insurance plan.

## Methods

We used data from eight cycles of the Canadian Community Health Survey (CCHS) conducted between 2007 and 2017. CCHS is an annual national survey that collects health-related information from Canadian residents to facilitate health research and inform program planning. The survey targets residents of Canada’s 13 provinces and territories aged 12 and above. CCHS does not include individuals living on Indian Reserves and on Crown Lands, institutional residents, full-time members of the Canadian Forces, and residents of certain remote regions. CCHS does not collect information on supplemental health insurance coverage on an annual basis in all provinces and territories. Only New Brunswick, Ontario, and Nunavut opted into the optional supplemental health insurance CCHS modules on more than a few occasions since the mid-2000s. We used data collected from New Brunswick residents in 2007, 2008, 2011, and 2013 to 2017; supplemental health insurance data were not collected in 2009, 2010, and 2012 (Statistics Canada, 2007, 2008, 2011, 2013, 2014, 2015, 2016, 2017).

We used as our outcome variables responses to the questions: Do you have insurance that covers all or part of the cost of your prescription medications? In 2007, 2008, 2011, 2013, and 2014, it had response categories: government-sponsored plan; employer-sponsored plan; private plan. Response categories from 2015 to 2017 were as follows: government-sponsored plan; employer-sponsored benefit plan; plan sponsored through an association such as a union, trade association or student organization; other, such as your own private plan purchased from an insurance company. Since most individuals who reported private coverage reported having an employer or association-sponsored plan, for ease of interpretation we combined all private coverage into a single category.

We used two ordinal measures of socioeconomic status (SES). First, we used a measure of household income in deciles (the ratio of household income to the low-income cut-off for the relevant household size and community size at the province level). In cases where respondents could not provide a specific amount, income was imputed by Statistics Canada. We included a binary variable for cases where income was imputed. To ease interpretation, we provide in the Appendix the mean and median household income (unadjusted) for each decile, by CCHS survey cycle (Appendix Table 7). Second, we used a measure of education (high school or less, some post-secondary below the bachelor’s level, bachelor’s degree or above). We adjusted for the following demographic characteristics: age, sex/gender, and linguistic identity (English, French, English and French, other). We included two measures of health: (1) self-reported health using responses to the question: “In general, would you say your health is excellent, very good, good, fair, or poor?” (respondents were prompted to consider physical, mental, and social health when giving their responses); and, (2) an indicator for self-reported chronic diseases if the respondent reported having been diagnosed with at least one of the following: high blood pressure, heart disease, cancer, diabetes, joint pain or arthritis, chronic lung problems, or mental health problems. Last, to explore the introduction of the New Brunswick Drug Plan, we (1) included binary indicators of the survey cycle; (2) included a binary NBDP indicator (i.e., pre-, post-May 2014); and (3) interacted the NBDP indicator with income (using four income categories, deciles 1, 2, 3–5, 6–10).

We used logistic regressions to examine characteristics associated with the odds of reporting supplementary drug insurance coverage. Since the parameters of non-linear models may be inconsistent in the presence of heteroskedasticity, we tested for its presence and rejected the null hypothesis of homoskedasticity. We then estimated maximum likelihood heteroskedastic probit models allowing all covariates to appear in the variance function and found, with one exception, no qualitatively important differences between these models and models that do not consider heteroskedasticity. We provide absolute and relative estimates of associations. First, we report odds ratios (OR) and 95% confidence intervals (the estimated ORs are conditional on all other explanatory variables). Estimated ORs obtained using different samples or with a different set of explanatory variables are not estimating the same parameters and should be compared with caution (Norton & Dowd, 2018). Second, we present predicted probabilities by socioeconomic characteristics (predicted probabilities calculated by setting each of the other covariates to their respective sample observed relative frequencies).

Since the NBPDP provides specific drug coverage to low-income seniors, we analyzed separately those 25–64 years and those ≥ 65 years of age. We excluded youth and young adults (12−24 years old) because they are most often covered by their parents’ drug plans. However, for those over age 24, we did not exclude any respondents in constructing figures of drug insurance coverage; those with non-response were included in the denominators. In our regression analyses, we used listwise deletion and dropped less than 5% of respondents. All analyses were conducted using Stata/SE 16.1 with the CCHS microdata Master files and Statistics Canada’s bootstrap weights.

## Results

We used responses from 9996 and 4909 New Brunswick residents aged 25 to 64 years and ≥ 65 years, respectively, with a small number reporting overall coverage but not public or private coverage separately.

### Socioeconomic status

**Fig. 2:**
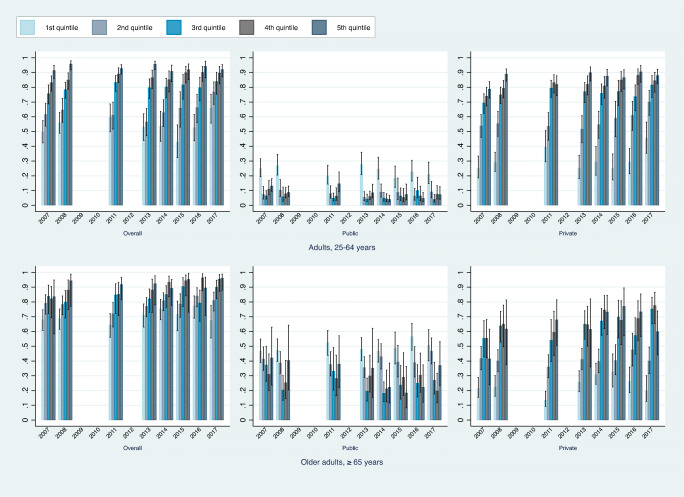
Self-reported drug coverage, by type, age group, and household income, New Brunswick, 2007–2017 (Source: Canadian Community Health Surveys, Statistics Canada)

From 2007 to 2017, lower-SES New Brunswick residents consistently reported having more public coverage but less private and overall coverage (Tables 1 and 2, Fig. 2, Appendix Fig. 4). For adults (25–64 years), the odds of reporting any—private or public—drug coverage were substantially higher among those who ranked in the 4th to 10th income deciles than among those who ranked in the 1st income decile. For example, those ranked in the 10th income decile had odds of reporting drug insurance coverage that were over seven times higher than those of respondents in the lowest decile (OR 7.3, 95% CI 4.7, 11.2). Adults in the 2nd to 10th income deciles had lower odds of reporting public drug insurance coverage and greater odds of reporting private drug insurance coverage than their peers in the lowest income decile. However, those in deciles 2 to 10 had more or less similar odds of having reported public drug coverage. Relative to adults in the first income decile, those in the 2nd decile had 0.25 times the odds of reporting public drug coverage (OR 0.25, 95% CI 0.17, 0.36) while those in the 10th decile had 0.2 times the odds of reporting public drug coverage (OR 0.20, 95% CI 0.14, 0.29). In contrast, there was a clear and steep gradient among adults who reported having private drug insurance. Adults in the top income decile had more than 14 times the odds of reporting private drug insurance relative to respondents in the 1st decile (OR 14.5, 95% CI 10.1, 20.9). Similar socioeconomic differences were observed in education attainment. For example, adults 25–64 with some post-secondary education below a bachelor’s degree and those with a bachelor’s degree or higher had higher odds of having reported drug insurance coverage than their peers with a high school education or less (OR 1.4, 95% CI 1.2, 1.7; OR 2.2, 95% CI 1.7, 3.0).

**Table 1:** Characteristics associated with the odds of reporting drug insurance coverage, New Brunswick, 2007–2017, 25–64 years old — year binary indicators

	All		Public		Private	
	OR	95% CI	OR	95% CI	OR	95% CI
Household income (ref: 1st decile, low)						
2nd decile	0.69**	0.51,0.93	0.25***	0.17,0.36	1.84***	1.32,2.57
3rd decile	0.98	0.74,1.30	0.21***	0.15,0.31	2.80***	2.06,3.81
4th decile	1.55***	1.15,2.09	0.20***	0.14,0.29	4.56***	3.31,6.28
5th decile	2.13***	1.59,2.84	0.15***	0.10,0.22	6.84***	5.01,9.34
6th decile	3.35***	2.45,4.58	0.16***	0.11,0.23	9.48***	6.88,13.06
7th decile	3.75***	2.67,5.28	0.18***	0.12,0.26	10.17***	7.25,14.27
8th decile	5.28***	3.61,7.71	0.21***	0.15,0.30	11.82***	8.34,16.74
9th decile	7.20***	4.82,10.75	0.31***	0.22,0.44	11.68***	8.22,16.59
10th decile, high	7.31***	4.76,11.23	0.20***	0.14,0.29	14.53***	10.10,20.91
Income imputed	0.87	0.73,1.04	0.69***	0.55,0.86	1.02	0.86,1.20
Education (ref: ≤ high school)						
Some post-secondary < bachelor’s level	1.40***	1.19,1.65	0.73***	0.59,0.90	1.61***	1.38,1.87
Bachelor’s degree or above	2.21***	1.65,2.95	0.93	0.71,1.21	2.16***	1.70,2.74
Self-reported health (ref: excellent/very good)						
Good	0.86*	0.72,1.03	0.88	0.71,1.09	0.92	0.78,1.08
Fair	0.97	0.73,1.28	1.66***	1.26,2.20	0.70***	0.55,0.90
Poor	1.30	0.90,1.89	2.55***	1.77,3.68	0.66**	0.47,0.94
Chronic diseases	1.02	0.86,1.21	1.86***	1.51,2.29	0.79***	0.67,0.92
Linguistic identity (ref: English)						
French	0.66***	0.57,0.78	0.95	0.79,1.15	0.69***	0.60,0.80
French & English	0.81	0.54,1.23	1.02	0.67,1.56	0.81	0.56,1.19
Other	2.00	0.31,13.02	17.70***	4.89,64.13	0.09***	0.02,0.36
Age (ref: 25–34)						
35–44	1.42***	1.12,1.79	1.00	0.75,1.33	1.39***	1.12,1.72
45–54	1.18	0.93,1.48	0.86	0.65,1.14	1.24*	1.00,1.54
55–64	1.23*	0.99,1.53	0.87	0.66,1.14	1.28**	1.04,1.57
Male	0.79***	0.68,0.92	1.00	0.83,1.20	0.85**	0.74,0.98
Year (ref: 2007)						
2008	1.28**	1.00,1.63	0.89	0.65,1.23	1.39***	1.11,1.74
2011	1.37**	1.05,1.78	0.87	0.62,1.22	1.49***	1.14,1.95
2013	1.17	0.90,1.52	0.76*	0.57,1.03	1.39***	1.09,1.77
2014	1.10	0.82,1.46	0.58***	0.42,0.81	1.41**	1.08,1.84
2015	1.17	0.86,1.59	0.66**	0.45,0.97	1.48***	1.11,1.99
2016	1.26	0.94,1.69	0.68**	0.48,0.97	1.61***	1.23,2.11
2017	1.75***	1.28,2.40	0.65**	0.45,0.92	2.21***	1.66,2.94
Constant	1.19	0.85,1.66	0.48***	0.33,0.69	0.26***	0.18,0.37
# of observations	9996		9981		9981	

Notes. *Household income*, ratio of household income to the low-income cut-off correspondent to the specific combination of household size and community size at province level; *chronic diseases*, if the respondent reported having been diagnosed with at least one of seven chronic diseases—high blood pressure, heart disease, cancer, diabetes, joint pain or arthritis, chronic lung problems like asthma or chronic obstructive pulmonary disease, and mental health problem; # of observations differs between models because a small number of respondents reported overall coverage but not public or private coverage separately; *, **, and ***, significant at 10%, 5%, and 1%, respectively

**Table 2:** Characteristics associated with the odds of reporting drug insurance coverage, New Brunswick, 2007–2017, ≥ 65 years old — year binary indicators

	All		Public		Private	
	OR	95% CI	OR	95% CI	OR	95% CI
Household income (ref: 1st decile, low)						
2nd decile	0.89	0.61,1.29	0.77	0.57,1.05	1.36*	0.95,1.95
3rd decile	1.31	0.89,1.94	0.75*	0.55,1.04	1.76***	1.23,2.53
4th decile	1.62**	1.07,2.47	0.39***	0.28,0.55	3.96***	2.74,5.73
5th decile	1.83**	1.13,2.97	0.34***	0.23,0.50	5.14***	3.45,7.65
6th decile	2.65***	1.54,4.58	0.24***	0.16,0.35	8.36***	5.47,12.77
7th decile	3.18***	1.78,5.70	0.35***	0.23,0.53	5.87***	3.81,9.06
8th decile	3.59***	1.84,6.98	0.35***	0.22,0.57	5.92***	3.65,9.58
9th decile	3.39***	1.67,6.91	0.53**	0.32,0.88	3.96***	2.36,6.64
10th decile, high	3.04**	1.27,7.27	0.36***	0.21,0.61	6.14***	3.47,10.88
Income imputed	1.11	0.88,1.38	0.73***	0.61,0.87	1.43***	1.18,1.73
Education (ref: ≤ high school)						
Some post-secondary < bachelor’s level	1.12	0.89,1.43	0.73***	0.61,0.88	1.61***	1.34,1.93
Bachelor’s degree or above	1.51*	0.98,2.33	0.57***	0.42,0.78	2.40***	1.77,3.25
Self-reported health (ref: excellent/very good)						
Good	1.03	0.81,1.30	1.19*	0.98,1.43	0.86	0.71,1.04
Fair	1.11	0.83,1.48	1.47***	1.17,1.84	0.70***	0.55,0.88
Poor	1.09	0.74,1.61	1.50**	1.07,2.12	0.82	0.55,1.21
Chronic diseases	1.49***	1.15,1.93	1.18	0.94,1.46	1.17	0.94,1.46
Linguistic identity (ref: English)						
French	0.59***	0.48,0.72	0.90	0.76,1.06	0.68***	0.57,0.81
French & English	1.04	0.57,1.88	1.33	0.79,2.24	0.67	0.38,1.19
Other	0.19	0.01,5.77	1.00	1.00,1.00	1.41	0.02,91.85
Age (ref: 65–74)						
75+	0.87	0.71,1.06	1.18**	1.00,1.39	0.77***	0.65,0.91
Male	0.91	0.74,1.11	0.93	0.79,1.10	1.04	0.88,1.23
Year (ref: 2007)						
2008	1.12	0.80,1.55	0.80	0.60,1.07	1.25	0.92,1.69
2011	1.11	0.78,1.58	1.08	0.79,1.47	0.96	0.70,1.33
2013	1.26	0.89,1.78	0.86	0.64,1.16	1.31*	0.95,1.80
2014	1.43**	1.01,2.02	0.83	0.62,1.12	1.43**	1.05,1.95
2015	1.46*	0.97,2.19	0.89	0.64,1.25	1.51**	1.06,2.14
2016	1.78***	1.18,2.67	0.86	0.63,1.19	1.73***	1.22,2.44
2017	1.41*	0.95,2.09	0.98	0.71,1.34	1.43**	1.03,1.98
Constant	2.06***	1.27,3.35	1.23	0.82,1.86	0.21***	0.13,0.33
# of observations	4909		4877		4879	

Notes. *Household income*, ratio of household income to the low-income cut-off correspondent to the specific combination of household size and community size at province level; *chronic diseases*, if the respondent reported having been diagnosed with at least one of seven chronic diseases—high blood pressure, heart disease, cancer, diabetes, joint pain or arthritis, chronic lung problems like asthma or chronic obstructive pulmonary disease, and mental health problem; # of observations differs between models because a small number of respondents reported overall coverage but not public or private coverage separately; *, **, and ***, significant at 10%, 5%, and 1%, respectively

The predicted probabilities of New Brunswick adult residents 25–54 in the lowest three income deciles reporting any drug coverage were 0.64 (95% CI 0.59, 0.69), 0.55 (95% CI 0.49, 0.61), and 0.63 (95% CI 0.59, 0.68), respectively. The same predicted probabilities for adult New Brunswickers in the top three income deciles were substantially higher at 0.90 (95% CI 0.88, 0.93), 0.93 (95% CI 0.91, 0.95), and 0.93 (95% CI 0.90, 0.95), respectively (Fig. 2, top panel). Differences in private coverage were even larger. The predicted probabilities for adult New Brunswickers reporting private drug coverage were 0.31 (95% CI 0.26, 0.36) for those in the lowest income decile and 0.87 (95% CI 0.84, 0.90) for those with the highest household income.

**Fig. 3:**
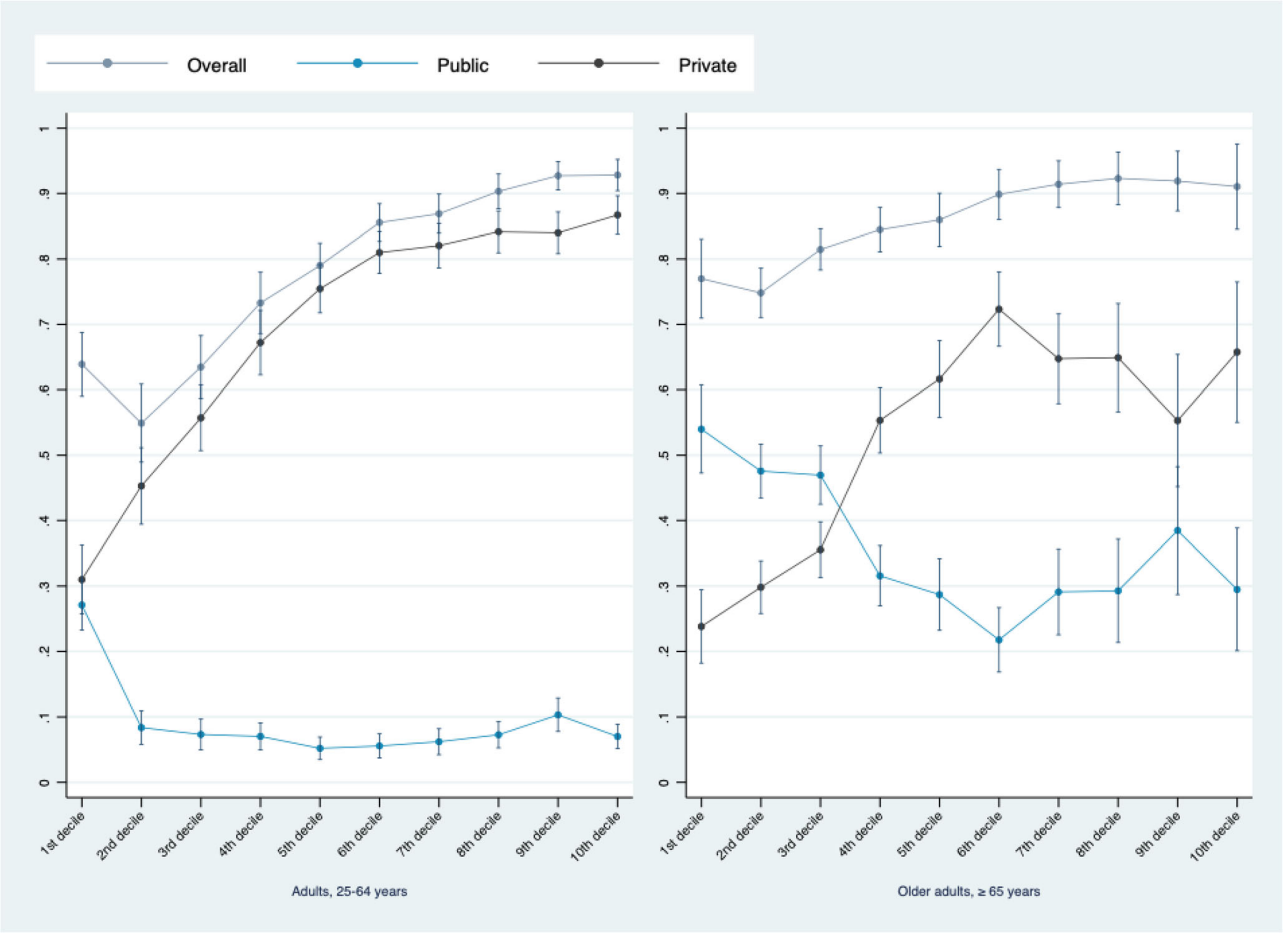
Predicted probability of having reported drug coverage, by type and age group, New Brunswick, 2007–2017

Similar socioeconomic gradients, albeit less steep, were also apparent among older adults (≥ 65 years) (Table 2). Older adults in the 7th, 8th, 9th, and 10th income deciles had odds of having reported drug coverage that were more than three times higher than those of the poorest older adults (OR 3.2, 95% CI 1.8, 5.7; OR 3.6, 95% CI 1.8, 7.0; OR 3.4, 95% CI 1.7, 6.9; OR 3.0, 95% CI 1.3, 7.3). These gradients were driven by the large differences in private coverage. For example, older adults in the 10th income decile of household income had substantially higher odds of having reported private drug coverage relative to their peers in the first decile (OR 6.1, 95% CI 3.5, 10.9). As was the case for those 25–54 years old, similar socioeconomic differences were observed in education attainment among older adults. Predictive probability estimates further highlight the association between income and drug insurance among older adults, particularly in private coverage. For example, the predicted probabilities for older adult New Brunswickers reporting private drug coverage were 0.24 (95% CI 0.18, 0.29) in the lowest income decile and 0.66 (95% CI 0.55, 0.76) for those with the highest household income (Fig. 3, right panel).

### Temporal trends

Adults (25–64 years) had significantly lower odds of having reported public coverage after the NBDP introduction in May 2014 (OR 0.73, 95% CI 0.61, 0.89 [Table 3]). However, reported public coverage showed a decline prior to 2014 and a stable profile subsequently (Table 1). Additionally, we found that income significantly modified the association between the NBDP introduction and public drug insurance; individuals with lower income (decile 1) had lower odds after NBDP was introduced, relative to before its implementation, while those in the 3rd to 5th income deciles had higher odds (Table 5). We found significantly higher odds of having reported private coverage after the NBDP introduction (OR 1.3, 95% CI 1.1, 1.5 [Table 3]). More specifically, we found an increase in the odds of private coverage between 2007 and 2008, a reasonably flat profile until increases from 2014 (Table 1). We did not find that income was an effect modifier (Table 5).

**Table 3:** Characteristics associated with the odds of reporting drug insurance coverage, New Brunswick, 2007–2017, 25–64 years old — New Brunswick Drug Plan (NBDP) binary indicator

	All		Public		Private	
	OR	95% CI	OR	95% CI	OR	95% CI
Household income (ref: 1st decile, low)						
2nd decile	0.70**	0.51,0.95	0.25***	0.17,0.36	1.85***	1.32,2.58
3rd decile	0.99	0.75,1.31	0.21***	0.15,0.31	2.80***	2.05,3.82
4th decile	1.55***	1.15,2.10	0.20***	0.14,0.29	4.53***	3.29,6.25
5th decile	2.11***	1.58,2.83	0.15***	0.10,0.22	6.74***	4.92,9.22
6th decile	3.35***	2.45,4.57	0.16***	0.11,0.23	9.40***	6.82,12.96
7th decile	3.71***	2.64,5.22	0.18***	0.12,0.26	9.97***	7.10,13.98
8th decile	5.23***	3.58,7.64	0.21***	0.15,0.30	11.64***	8.22,16.48
9th decile	7.13***	4.78,10.65	0.31***	0.22,0.44	11.47***	8.08,16.28
10th decile, high	7.18***	4.68,11.02	0.20***	0.14,0.29	14.16***	9.84,20.38
Income imputed	0.84**	0.71,0.99	0.68***	0.54,0.84	0.98	0.84,1.16
Education (ref: ≤ high school)						
Some post-secondary < bachelor’s level	1.39***	1.18,1.65	0.73***	0.59,0.90	1.60***	1.38,1.87
Bachelor’s degree or above	2.22***	1.65,2.98	0.93	0.71,1.21	2.17***	1.70,2.76
Self-reported health (ref: excellent/very good)						
Good	0.86*	0.72,1.03	0.87	0.70,1.08	0.92	0.78,1.08
Fair	0.97	0.73,1.28	1.66***	1.26,2.19	0.70***	0.55,0.90
Poor	1.28	0.89,1.86	2.52***	1.74,3.63	0.66**	0.47,0.93
Chronic diseases	1.02	0.86,1.21	1.85***	1.51,2.28	0.79***	0.67,0.92
Linguistic identity (ref: English)						
French	0.66***	0.57,0.78	0.95	0.79,1.15	0.69***	0.60,0.80
French & English	0.82	0.54,1.24	1.01	0.66,1.53	0.83	0.57,1.21
Other	2.08	0.35,12.41	17.97***	4.93,65.54	0.10***	0.03,0.37
Age (ref: 25–34)						
35–44	1.39***	1.10,1.76	1.00	0.75,1.33	1.37***	1.10,1.70
45–54	1.16	0.92,1.48	0.86	0.65,1.14	1.23*	0.99,1.54
55–64	1.22*	0.98,1.52	0.86	0.66,1.13	1.28**	1.04,1.58
Male	0.79***	0.67,0.92	1.00	0.83,1.20	0.85**	0.73,0.98
NBDP (ref: < May 1, 2014)	1.08	0.93,1.27	0.73***	0.61,0.89	1.27***	1.10,1.47
Constant	1.45**	1.08,1.95	0.42***	0.31,0.58	0.35***	0.25,0.48
# of observations	9996		9981		9981	

Notes. *Household income*, ratio of household income to the low-income cut-off correspondent to the specific combination of household size and community size at province level; *chronic diseases*, if the respondent reported having been diagnosed with at least one of seven chronic diseases—high blood pressure, heart disease, cancer, diabetes, joint pain or arthritis, chronic lung problems like asthma or chronic obstructive pulmonary disease, and mental health problem; # of observations differs between models because a small number of respondents reported overall coverage but not public or private coverage separately; *, **, and ***, significant at 10%, 5%, and 1%, respectively

Relative to 2007, older adults surveyed from 2014 to 2017 had odds of reporting any drug coverage that were about 40% higher (Tables 2 and 4). These associations were driven by an increase in the odds of reporting private drug coverage. Between 2014 and 2017, older adults had odds of reporting private drug coverage that were between about 40% and 75% higher than the odds of reporting the same coverage in 2007 (Table 5). These differences were driven by individuals in the second and sixth to tenth deciles (Table 6). We did not find any temporal differences in reporting public drug insurance among older adults.

**Table 4:** Characteristics associated with the odds of reporting drug insurance coverage, New Brunswick, 2007–2017, ≥ 65 years old — New Brunswick Drug Plan (NBDP) binary indicator

	All		Public		Private	
	OR	95% CI	OR	95% CI	OR	95% CI
Household income (ref: 1st decile, low)						
2nd decile	0.90	0.62,1.31	0.77*	0.57,1.04	1.37*	0.96,1.96
3rd decile	1.32	0.89,1.96	0.75*	0.54,1.03	1.78***	1.24,2.55
4th decile	1.63**	1.07,2.48	0.39***	0.28,0.55	3.97***	2.75,5.73
5th decile	1.86**	1.15,3.02	0.34***	0.23,0.50	5.21***	3.51,7.74
6th decile	2.66***	1.54,4.61	0.24***	0.16,0.35	8.35***	5.48,12.74
7th decile	3.23***	1.80,5.79	0.35***	0.23,0.53	5.94***	3.86,9.14
8th decile	3.63***	1.86,7.11	0.36***	0.22,0.57	5.88***	3.64,9.50
9th decile	3.41***	1.67,6.98	0.54**	0.33,0.88	3.95***	2.36,6.61
10th decile, high	3.08**	1.29,7.35	0.36***	0.21,0.62	6.16***	3.48,10.91
Income imputed	1.10	0.88,1.36	0.72***	0.60,0.86	1.42***	1.18,1.71
Education (ref: ≤ high school)						
Some post-secondary < bachelor’s level	1.12	0.88,1.42	0.73***	0.61,0.88	1.60***	1.33,1.93
Bachelor’s degree or above	1.51*	0.99,2.32	0.57***	0.42,0.78	2.41***	1.79,3.26
Self-reported health (ref: excellent/very good)						
Good	1.03	0.81,1.31	1.18*	0.98,1.42	0.86	0.72,1.04
Fair	1.11	0.83,1.49	1.47***	1.17,1.84	0.70***	0.55,0.88
Poor	1.09	0.74,1.62	1.48**	1.05,2.08	0.83	0.56,1.21
Chronic diseases	1.49***	1.14,1.94	1.18	0.95,1.48	1.17	0.94,1.46
Linguistic identity (ref: English)						
French	0.59***	0.48,0.72	0.90	0.76,1.06	0.68***	0.57,0.81
French & English	1.06	0.59,1.93	1.32	0.78,2.22	0.69	0.39,1.20
Other	0.17	0.01,5.14	1.00	1.00,1.00	1.24	0.02,78.29
Age (ref: 65–74)						
75+	0.87	0.71,1.07	1.18**	1.00,1.39	0.78***	0.66,0.92
Male	0.91	0.74,1.11	0.93	0.79,1.09	1.05	0.89,1.23
NBDP (ref: < May 1, 2014)	1.40***	1.14,1.71	0.98	0.83,1.15	1.37***	1.16,1.61
Constant	2.28***	1.46,3.56	1.14	0.79,1.64	0.23***	0.16,0.35
# of observations	4909		4877		4879	

Notes. *Household income*, ratio of household income to the low-income cut-off correspondent to the specific combination of household size and community size at province level; *chronic diseases*, if the respondent reported having been diagnosed with at least one of seven chronic diseases—high blood pressure, heart disease, cancer, diabetes, joint pain or arthritis, chronic lung problems like asthma or chronic obstructive pulmonary disease, and mental health problem; # of observations differs between models because a small number of respondents reported overall coverage but not public or private coverage separately; *, **, and ***, significant at 10%, 5%, and 1%, respectively

**Table 5:** Characteristics associated with the odds of reporting drug insurance coverage, New Brunswick, 2007–2017, 25–64 years old — New Brunswick Drug Plan (NBDP) binary indicator × income

	All		Public		Private	
	OR	95% CI	OR	95% CI	OR	95% CI
Household income (ref: 1st decile, low)						
2nd decile	0.59***	0.41,0.86	0.22***	0.13,0.35	1.94***	1.26,2.97
3rd–5th decile	1.21	0.91,1.59	0.15***	0.11,0.20	4.45***	3.14,6.30
6th–10th decile, high	4.09***	3.03,5.51	0.21***	0.16,0.27	10.91***	7.66,15.53
Income imputed	0.81**	0.69,0.96	0.67***	0.54,0.83	0.96	0.82,1.13
Education (ref: ≤ high school)						
Some post-secondary < bachelor’s level	1.45***	1.23,1.71	0.74***	0.60,0.90	1.64***	1.41,1.91
Bachelor’s degree or above	2.44***	1.83,3.26	0.96	0.74,1.24	2.31***	1.83,2.92
Self-reported health (ref: excellent/very good)						
Good	0.82**	0.69,0.98	0.86	0.69,1.07	0.89	0.75,1.04
Fair	0.92	0.70,1.22	1.65***	1.25,2.17	0.68***	0.53,0.87
Poor	1.17	0.81,1.69	2.40***	1.67,3.46	0.62***	0.44,0.87
Chronic diseases	1.01	0.86,1.20	1.87***	1.52,2.30	0.79***	0.67,0.92
Linguistic identity (ref: English)						
French	0.66***	0.56,0.77	0.95	0.78,1.14	0.69***	0.60,0.80
French & English	0.82	0.54,1.24	0.99	0.66,1.50	0.83	0.57,1.21
Other	1.74	0.26,11.55	18.86***	4.88,72.84	0.08***	0.02,0.34
Age (ref: 25–34)						
35–44	1.41***	1.12,1.78	1.02	0.77,1.36	1.37***	1.11,1.70
45–54	1.22*	0.96,1.54	0.87	0.66,1.15	1.27**	1.02,1.58
55–64	1.26**	1.01,1.57	0.88	0.67,1.16	1.30**	1.05,1.60
Male	0.80***	0.69,0.94	1.01	0.84,1.21	0.86**	0.74,0.99
NBDP (ref: < May 1, 2014)	0.80	0.54,1.18	0.64**	0.44,0.92	1.28	0.80,2.04
Income × NBDP						
2nd decile × NBDP	1.38	0.74,2.56	1.37	0.64,2.94	0.90	0.46,1.75
3rd–5th decile × NBDP	1.54*	0.96,2.45	1.71**	1.01,2.89	0.98	0.58,1.67
6th–10th decile × NBDP	1.32	0.83,2.11	1.02	0.64,1.62	0.99	0.59,1.66
Constant	1.68***	1.22,2.31	0.44***	0.32,0.62	0.35***	0.24,0.50
# of observations	9996		9981		9981	

Notes. *Household income*, ratio of household income to the low-income cut-off correspondent to the specific combination of household size and community size at province level; *chronic diseases*, if the respondent reported having been diagnosed with at least one of seven chronic diseases—high blood pressure, heart disease, cancer, diabetes, joint pain or arthritis, chronic lung problems like asthma or chronic obstructive pulmonary disease, and mental health problem; # of observations differs between models because a small number of respondents reported overall coverage but not public or private coverage separately; *, **, and ***, significant at 10%, 5%, and 1%, respectively

**Table 6:** Characteristics associated with the odds of reporting drug insurance coverage, New Brunswick, 2007–2017, ≥ 65 years old — New Brunswick Drug Plan (NBDP) binary indicator × income

	All		Public		Private	
	OR	95% CI	OR	95% CI	OR	95% CI
Household income (ref: 1st decile, low)						
2nd decile	0.61**	0.42,0.89	0.67**	0.48,0.95	0.93	0.60,1.44
3rd–5th decile	1.07	0.74,1.54	0.47***	0.34,0.65	2.41***	1.62,3.57
6th–10th decile, high	2.07***	1.32,3.25	0.41***	0.28,0.59	4.13***	2.69,6.35
Income imputed	1.09	0.88,1.35	0.72***	0.61,0.86	1.40***	1.17,1.68
Education (ref: ≤ high school)						
Some post-secondary < bachelor’s level	1.13	0.90,1.43	0.71***	0.59,0.85	1.63***	1.36,1.95
Bachelor’s degree or above	1.58**	1.02,2.45	0.58***	0.43,0.78	2.42***	1.81,3.25
Self-reported health (ref: excellent/very good)						
Good	1.03	0.81,1.30	1.19*	0.99,1.43	0.85*	0.70,1.03
Fair	1.12	0.84,1.49	1.49***	1.19,1.87	0.70***	0.55,0.87
Poor	1.09	0.74,1.62	1.53**	1.09,2.14	0.78	0.53,1.15
Chronic diseases	1.51***	1.16,1.95	1.13	0.91,1.42	1.21*	0.97,1.51
Linguistic identity (ref: English)						
French	0.58***	0.48,0.71	0.90	0.77,1.07	0.68***	0.57,0.80
French & English	1.06	0.59,1.93	1.27	0.76,2.13	0.71	0.40,1.26
Other	0.15	0.01,4.39	1.00	1.00,1.00	1.08	0.05,24.27
Age (ref: 65–74)						
75+	0.86	0.71,1.06	1.18**	1.01,1.39	0.77***	0.65,0.92
Male	0.91	0.74,1.11	0.94	0.80,1.10	1.03	0.88,1.22
NBDP (ref: < May 1, 2014)	0.74	0.41,1.35	0.92	0.55,1.53	0.82	0.45,1.49
Income × NBDP						
2nd decile × NBDP	2.12**	1.04,4.30	1.31	0.72,2.39	2.04**	1.00,4.13
3rd–5th decile × NBDP	1.95**	1.01,3.77	1.19	0.68,2.10	1.45	0.76,2.76
6th–10th decile × NBDP	2.16*	0.99,4.68	0.74	0.41,1.35	2.03**	1.03,4.00
Constant	3.19***	2.04,4.98	1.21	0.83,1.76	0.31***	0.20,0.47
# of observations	4909		4877		4879	

Notes. *Household income*, ratio of household income to the low-income cut-off correspondent to the specific combination of household size and community size at province level; *chronic diseases*, if the respondent reported having been diagnosed with at least one of seven chronic diseases—high blood pressure, heart disease, cancer, diabetes, joint pain or arthritis, chronic lung problems like asthma or chronic obstructive pulmonary disease, and mental health problem; # of observations differs between models because a small number of respondents reported overall coverage but not public or private coverage separately; *, **, and ***, significant at 10%, 5%, and 1%, respectively

### Self-reported health status and chronic diseases

Overall, there were no discernible differences in the reporting of drug coverage between New Brunswickers of differing self-reported health statuses. There were, however, stark differences in public and private coverage. Adults and older adults who reported lower health status had generally lower odds of reporting private drug coverage but higher odds of reporting public drug coverage (Tables 1 and 2). For example, relative to adults who reported very good or excellent health, those who reported poor health had more than twice the odds of also reporting public drug coverage (OR 2.6, 95% CI 1.8, 3.7) but substantially lower odds of reporting private drug coverage (OR 0.7, 95% CI 0.5, 0.9). We found similar associations among adults who reported having at least one chronic disease but not among older adults who had higher odds of having reported any drug coverage (OR 1.5, 95% CI 1.2, 1.9).

### Linguistic identity

Relative to anglophones, francophones were less likely to report any drug coverage. This was the case for adults and older adults (OR 0.7, 95% CI 0.6, 0.8; OR 0.6, 95% CI 0.5, 0.7). These associations were driven by differences in private coverage. Francophone adults and older adults had lower odds of having reported private drug coverage (OR 0.7, 95% CI 0.6, 0.8; OR 0.7, 95% CI 0.6, 0.8). Although not precisely estimated, relative to anglophones, we found that adults who were neither anglophones nor francophones had higher odds of having public coverage (OR 17.7, 95% CI 4.9, 64.1) and lower odds of having reported private coverage (OR 0.09, 95% CI 0.02, 0.36).

### Sex/gender

We found that adult males had lower odds of reporting private drug insurance than adult females (OR 0.85, 95% CI 0.74, 0.98) but not public drug insurance (OR 1.00, 95% CI 0.83, 1.20). We did not find any statistically significant or meaningful sex/gender differences among older adults in the reporting of drug insurance (public or private).

## Discussion

We found statistically significant and substantial socioeconomic differences in the reporting of prescription drug insurance coverage among adults and older adults in New Brunswick; differences were large enough to be policy meaningful. Individuals in the second decile of household income were particularly vulnerable to reporting neither public nor private drug coverage. For example, adults in the second income decile had predicted probabilities of having reported any drug coverage that were 14 percentage points lower than those of individuals in the lowest income decile and 38 percentage points lower than those of individuals in the top income decile. These results are consistent in direction with, but somewhat larger in magnitude than, studies that documented socioeconomic differences in the reporting of drug insurance coverage nationally, as well as in British Columbia, Ontario, and Québec since the late 1990s (Allin & Hurley, 2009; Barnes et al., 2015; Dewa et al., 2005; Guo et al., 2020; Kapur & Basu, 2005).

The introduction of the New Brunswick Drug Plan in 2014 does not appear to have led to more New Brunswick residents reporting public drug coverage; however, from 2014 the decreasing trend in public drug coverage appears to have ceased. Additionally, the increasing trend in private coverage from 2014 could potentially be explained by the misreporting of the type of drug insurance coverage, as has been observed among older adults in Ontario (Guo et al., 2020).

Although we did not find many differences in the reporting of drug coverage between individuals with differing self-reported health statuses, we found that those who reported lower health status had generally lower odds of reporting private drug coverage but higher odds of reporting public drug coverage. These results are not surprising as they reflect, at least in part, the design of the New Brunswick drug insurance system whereby the sick and poor are covered by public insurance while private insurers target the wealthy and healthy, particularly in the individual market. In the employer-based group market, it may be the case that employers that offer drug benefits tend to have healthier employees. Last, differences in health may be associated with the misreporting of one’s drug insurance coverage.

We also found that relative to anglophones, francophones were less likely to report any drug coverage, and this was driven by differences in private coverage. We do not have a good explanation for this finding besides pointing out that differences in employment between francophones and anglophones may explain some of the differences in drug coverage (Desjardins & Campbell, 2019; Lepage, 2012). Adjusting for employment (not shown), however, did not change this finding in any meaningful way.

### Limitations

First, our dependent variables represent self-reported drug coverage which may differ from actual drug coverage. There is some evidence that CCHS respondents misreport drug coverage. For example, in 2015–2016, about 50% of older Ontarians (≥ 65 years) reported no public coverage and more than 20% reported having no coverage at all. However, nearly all older adults in Ontario qualify for the publicly funded Ontario Drug Benefit (ODB) Program (Guo et al., 2020). It is important to note that being unaware of one’s insurance coverage may also hinder access to essential drugs. Second, the repeated cross-sectional data we used do not allow us to make any causal claims about the effects of SES, health status, linguistic identity, or the introduction of the NBDP on the reporting of drug coverage. In particular, we had only four periods of data before and after the policy change. This limited our ability to use quasi-experimental approaches, such as interrupted-time-series designs, that are often used to evaluate causal relationships or associations between health policies, drug use, and health outcomes (Jandoc et al., 2015; Lopez Bernal et al., 2017). Third, we found one meaningful difference between estimates obtained using logistic and heteroskedastic probit models. The estimates for the associations between linguistic identity and reporting drug insurance coverage for those categorized as “other” relative to anglophones varied substantially in magnitude (although not in direction) and should be interpreted with caution. Last, Statistics Canada implemented changes to the CCHS methodology (survey frame, sampling, collection, and weighting) and, as a result, cautions about making comparisons between cycles conducted before and after 2015. Using data from CCHS’s 2007, 2008, 2011, 2013, and 2014 cycles, we did not find any qualitatively important differences in our results.

## Conclusion

Our findings add to existing research that documented inequities in insurance coverage for prescription drugs in Canada and raise broader questions about the approach to pharmacare actually adopted in New Brunswick (Allin & Hurley, 2009; Barnes et al., 2015; Dewa et al., 2005; Guo et al., 2020; Kapur & Basu, 2005). In particular, the introduction of a public option in 2014, the New Brunswick Drug Plan, does not appear to have substantially reduced socioeconomic differences in drug insurance coverage. In addition to focusing on the poorest, there is also a need to address the considerable lack of coverage reported by individuals in the 2nd and 3rd household income deciles. In light of the evidence which indicates that public health insurance is generally more efficient than insurance plans that are privately provided, and that the provision of private health insurance in Canada had become increasingly less efficient between 1991 and 2011, New Brunswick’s increasing reliance on private drug insurance, especially for those over age 65, is of concern and warrants additional research (Himmelstein et al., 2020; Hurley & Guindon, 2020; Kratzer et al., 2013; Law et al., 2014; Morgan et al., 2017). Finally, our finding that francophones, relative to anglophones, were less likely to report any drug coverage deserves closer examination.

## Contributions to knowledge

### What does this study add to existing knowledge?

- Our findings add to existing research that documented inequities in insurance coverage for prescription drugs in Canada.
- In New Brunswick, between 2007 and 2017, we found statistically significant, substantial and policy-relevant socioeconomic differences in the reporting of prescription drug insurance coverage among those 25–64 years and those ≥ 65 years of age, and an increasing reliance on private drug insurance over time.
- The introduction of a public option in 2014, the New Brunswick Drug Plan, does not appear to have substantially reduced socioeconomic differences in drug insurance coverage.

### What are the key implications for public health interventions, practice, or policy?

- In light of the evidence which indicates that public health insurance is generally more efficient than insurance plans that are privately provided, and that the provision of private health insurance in Canada had become increasingly less efficient between 1991 and 2011, New Brunswick’s increasing reliance on private drug insurance, especially for those over age 65, is of concern.

## Figures and Tables

**Fig. 4:**
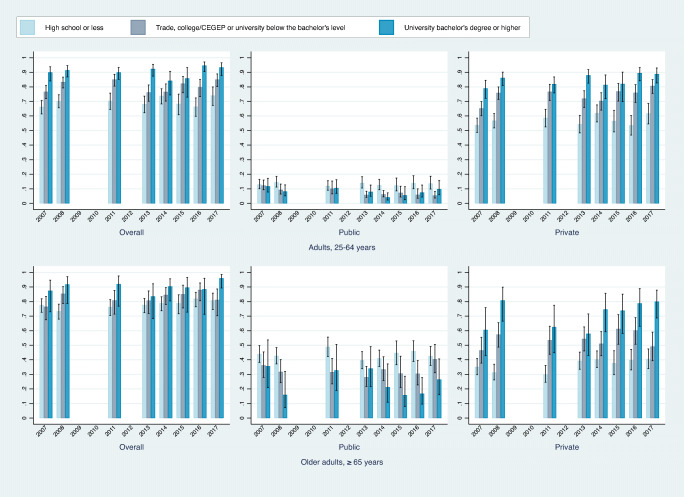
Self-reported drug coverage, by type, age group and education, New Brunswick, 2007–2017

## Data Availability

All data are available to qualified researchers at Statistics Canada Research Data Centres.

## Appendix

**Table 7:** New Brunswick, household income deciles, mean and median, by Canadian Community Health Survey cycle

Decile:	1	2	3	4	5	6	7	8	9	10
Mean										
2007	12,477	21,168	30,414	37,934	48,366	56,038	62,034	77,176	93,087	143,567
2008	12,311	21,506	29,101	39,824	47,228	54,723	65,487	80,055	96,822	145,969
2011	15,573	24,675	32,400	39,404	51,784	60,800	78,724	88,798	101,777	161,343
2013	14,920	25,156	32,325	43,414	50,425	63,773	77,301	90,775	110,061	178,429
2014	15,305	25,338	33,018	43,393	52,201	62,105	76,689	86,897	110,384	170,004
2015	16,300	26,913	31,975	42,309	56,621	65,430	77,720	95,150	113,618	369,542
2016	16,516	31,292	44,461	58,602	67,854	78,062	92,242	113,815	138,680	197,854
2017	17,994	32,678	45,321	58,312	71,003	86,385	98,721	121,834	134,317	231,473
Median										
2007	12,000	20,000	28,000	35,000	45,000	51,000	60,000	75,000	85,000	130,000
2008	11,000	20,000	27,600	38,000	45,000	50,000	64,000	75,000	90,000	130,000
2011	15,000	24,000	30,000	40,000	50,000	58,000	80,000	90,000	95,000	150,000
2013	14,000	24,000	30,000	40,000	49,200	62,000	75,000	87,000	100,000	150,000
2014	13,680	24,000	30,000	40,000	50,000	60,000	70,000	80,000	100,000	150,000
2015	16,000	24,000	30,000	40,000	50,000	60,000	72,000	90,000	110,000	160,000
2016	16,222	29,243	40,000	54,053	64,475	78,803	87,710	102,230	133,892	183,138
2017	18,280	29,782	41,057	53,556	65,568	81,995	95,300	116,105	130,000	192,103

## Code availability

Stata code is available upon request from the corresponding author.
